# Translating theory into practice: students' lived experiences on the utilization of OSVE and role-play for acquiring psychiatric nursing competencies

**DOI:** 10.25122/jml-2024-0383

**Published:** 2025-04

**Authors:** Amal Ibrahim Khalil, Samirh Said Alqhtani

**Affiliations:** 1 Nursing Department, College of Nursing, King Saud bin Abdulaziz University for Health Sciences, Jeddah, Saudi Arabia; 2 King Abdullah International Medical Research Center, Jeddah, Saudi Arabia; 3 Psychiatric and Mental Health Nursing, Faculty of Nursing, Menoufia University, Shebin El-Kom, Egypt; 4 Nursing Department, College of Nursing, King Saud Bin Abdulaziz University for Health Sciences, Riyadh, Saudi Arabia

**Keywords:** Psychiatric nursing education, objective structured video examination (OSVE), role-play, nursing competencies, lived experiences, skills acquisition, qualitative study, student perceptions, clinical reasoning, and psychiatric nursing skills

## Abstract

Developing key competencies like communication, empathy, and critical decision-making is crucial in psychiatric nursing education to prepare students for complex clinical environments. This study explored nursing students' experiences in using objective structured video examination (OSVE) and role-play to develop essential psychiatric nursing competencies. A qualitative approach was used, gathering data through focus group discussions and individual interviews with students in a psychiatric nursing course. Various themes emerged, highlighting the advantages and limitations of each teaching method. OSVE offered structured scenarios that improved clinical reasoning and self-assessment, while role-play encouraged real-time interactions and adaptive thinking. Students found that OSVE helped them connect theoretical knowledge to practical applications by allowing them to observe and analyze model behaviors in different psychiatric situations. On the other hand, role-play promoted deeper engagement through realistic patient interactions, boosting confidence and emotional readiness. However, students also faced challenges like performance anxiety during role-play and the need for guidance in OSVE sessions. This study emphasizes the complementary nature of OSVE and role-play in enhancing psychiatric nursing competencies and suggests integrating both methods for comprehensive skill development. The findings offer valuable insights for educators to enhance teaching strategies in psychiatric nursing education, aligning with evidence-based practices to meet student learning needs.

## INTRODUCTION

Integrating simulation-based education, such as objective structured clinical examinations (OSCE) and role-play, is essential in training psychiatric nursing students to bridge the gap between theory and practice. These methods enhance students' clinical competencies, especially in communication, empathy, and therapeutic interaction, which are crucial in psychiatric care.

Experiential learning techniques like objective structured video examinations (OSVE) and role-playing are integrated into psychiatric nursing education to prepare students for the dynamic nature of practice. OSVE uses video simulations to improve diagnostic reasoning and decision-making skills, while role-playing enhances empathy and communication abilities [1].

Psychiatric nursing education has been strengthened to meet the demands of modern healthcare by integrating experiential learning techniques, such as OSVE and role-playing, into the curriculum. Traditional teaching methods, which are primarily focused on theoretical instruction, often fail to adequately prepare students for the dynamic and interpersonal nature of psychiatric nursing practice. Consequently, innovative pedagogical strategies are adopted to enhance students’ clinical competencies, especially in areas like communication, empathy, and therapeutic interaction, which are essential in psychiatric care [1,2].

OSVE, which utilizes video simulations of clinical scenarios, enables nursing students to conduct structured reflection and critical analysis of psychiatric cases. This method has been shown to improve diagnostic reasoning, therapeutic communication, and decision-making skills, providing a risk-free environment where students can practice before engaging with real patients [3]. Role-playing is another widely utilized technique in psychiatric nursing education that allows students to simulate real-life interactions, developing their empathy and interpersonal communication skills [4]. Research indicates that role-playing can significantly enhance students' confidence, critical thinking, and problem-solving abilities in the psychiatric setting [5,6].

While the benefits of OSVE and role-playing are well documented, understanding students' perspectives on these methods in psychiatric nursing education is crucial for refining teaching approaches. Research shows that simulation-based education improves clinical reasoning, critical thinking, and decision-making skills in nursing students.

Moreover, the use of simulated patients and role-play helps bridge the theory-to-practice gap by allowing students to apply their knowledge in real-life scenarios. Various studies have endorsed this approach as a vital component of undergraduate nursing curricula, especially in mental health education [7]. A study by Labrague *et al*. [8] found that nursing students who participated in simulation-based education demonstrated better clinical reasoning, critical thinking, and decision-making skills than those who received traditional classroom-based instruction [9]. Furthermore, research has highlighted the benefits of integrating OSCE and role-play in psychiatric nursing education. A study by Poore *et al*. [10] revealed that OSCE scenarios focusing on mental health assessment and intervention skills improved students' confidence and competence in managing psychiatric patients [11]. Similarly, a study by Görücü, Türk, and Karaçam [12] demonstrated that role-play with standardized patients (SPs) enhanced nursing students' communication skills, empathy, and therapeutic relationships with mental health clients. Exploring students' perspectives is vital for refining these educational approaches and ensuring they effectively prepare students for clinical practice. Simulation-based learning, including OSVE and role-playing, has been linked to improved self-efficacy and clinical competency across various domains of nursing practice [13,14], yet specific studies focusing on psychiatric nursing are limited [15].

This qualitative study explored nursing students' perceptions of OSVE and role-play in acquiring essential psychiatric nursing skills. By understanding their experiences, the research aims to enhance the development of core competencies in psychiatric care and improve their application in nursing education.

### Significance of the study

The significance of this research lies in bridging the gap between theoretical knowledge and practical skills in psychiatric nursing. Understanding how students comprehend and encounter essential competencies in managing psychiatric symptoms can improve curricula and instructional approaches, leading to better patient outcomes and increased mental health awareness.

This investigation plays a vital role in enhancing nursing education and psychiatric care by examining how undergraduate students comprehend and encounter the instruction of essential competencies related to managing psychiatric symptoms, including hallucinations, delusions, suicidal ideation, aggression, and anxiety. The results can aid in improving curricula and instructional approaches, better equipping nurses to navigate the intricacies of psychiatric care. By enhancing symptom management abilities, the study contributes to improved patient safety, increased mental health awareness, reduced stigmatization, and patient-centered care. Its broader influence extends to healthcare quality, fostering improved patient outcomes and encouraging ongoing research and professional growth in psychiatric nursing.

### Conceptual framework

#### Integration of Benner's novice to expert theory and constructivism

This study combines Benner's novice to expert theory and constructivism to provide a comprehensive conceptual framework for understanding nursing education. Benner's theory outlines the progression from novice to expert in nursing, emphasizing experiential learning and mentorship [16]. Constructivism highlights the active role of nursing students in constructing knowledge through their educational experiences. Simultaneously, constructivism serves as a foundational perspective that underscores the active role of nursing students in constructing knowledge and understanding through their educational experiences [17]. In this framework, nursing students engage in clinical contexts, simulations, and collaborative interactions with peers and instructors. They reflect on these experiences, integrate new knowledge, and adapt their approaches to symptom management, aligning with constructivist principles [18].

By integrating these theories, the study explored how nursing students develop competencies in symptom management, particularly in psychiatric nursing, considering their educational experiences, progression, and professional development. This dual framework offers a holistic perspective on nursing students' journey from novice to expert and the active construction of knowledge in their educational experiences. This integrated framework aligns the progressive development of nursing competence with active knowledge construction, providing a holistic perspective for the study's exploration of nursing students' experiences in psychiatric symptom management.

### Aim of the study

This study explored students' experiences in psychiatric nursing using OSVE and role-play as learning methods. It aimed to understand how these methods help students develop therapeutic communication, empathy, and clinical decision-making skills. The study also investigated students' perceptions of how effectively these tools help them apply theoretical knowledge in real psychiatric care settings. Additionally, it evaluated the advantages and challenges students encounter when using these simulation-based techniques in their education.

## MATERIAL AND METHODS

### Study design

This study follows an inductive qualitative phenomenology design, guided by the conceptual frameworks of phenomenology, Benner's novice to expert theory, and constructivism. Phenomenology aims to understand the meaning of participants' lived experiences [19], while constructivism explores phenomena from the participants' perspectives in teaching and learning activities [20]. Benner's theory illustrates students' progression from novice to expert in nursing education, focusing on competence and expertise development in psychiatric nursing [21]. This framework helps understand how nursing students acquire skills in symptom management and their educational journey. The qualitative methodology was chosen for its ability to provide rich insights into student nurses' experiences. This design allowed for a deeper understanding of the challenges faced by undergraduate nursing students in their clinical training in psychiatric nursing [22]. By following this design, we gained insights into the challenges faced by undergraduate nursing students at the College of Nursing during their clinical training in the psychiatric nursing course.

### Study sample

The study involved nursing students in a psychiatric mental health course during the 3^rd^ semester of the 2022–2023 academic year. Purposeful sampling was used to select participants who could provide in-depth insights into the phenomenon under investigation [23]. The participants were divided into four groups, each containing five students, based on their experiences in psychiatric clinical courses, either OSVE or role-play simulation. The sample size in the qualitative study was determined based on data saturation [23]. Data saturation was achieved after analyzing data from 20 participants.

The inclusion criteria were students who attended most competencies and OSVE sessions, utilized role-play, agreed to participate in the study, and were enrolled in a psychiatric mental health course at the College of Nursing in Jeddah. The exclusion criteria were students not enrolled in a psychiatric mental health course and nursing students from different universities.

### Data collection procedure

**Figure 1:**
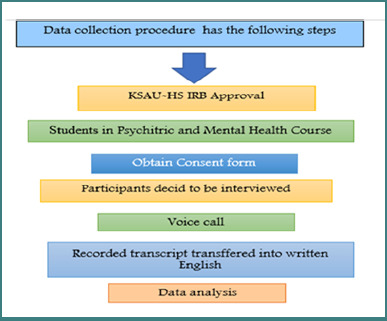
Flowchart of the qualitative data collection and analysis process

After receiving IRB approval from King Saud bin Abdulaziz University for Health Sciences (KSAU-HS), the researchers contacted nursing students through the student academic affairs departments. The study's aim was explained to the participants, and any questions regarding the study or the consent form were addressed. The researchers scheduled interviews with participants based on their preferences and availability. Consent was obtained either verbally or via email, with electronic signatures (Figure 1).

### Intervention design and procedure

**Figure 2:**
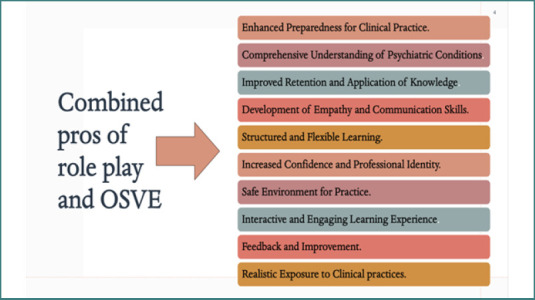
Combined benefits of role-play and OSVE for psychiatric nursing competency development

This study used two interactive teaching strategies to improve psychiatric nursing competencies: role-playing and the OSVE (Figure 2). All students participated in the study's eight competency-based sessions under uniform guidelines. OSVE sessions were crafted to bolster students' capabilities in diagnosing and assessing psychiatric patients. These sessions incorporated pre-recorded video scenarios, either sourced from platforms such as YouTube or produced by the course instructors. The latter involved instructors portraying simulated patients exhibiting various psychiatric conditions, including schizophrenia, bipolar disorder, and personality disorders. Each OSVE scenario, meticulously designed to mirror authentic clinical interactions, spanned approximately 20–25 minutes.

After viewing the OSVE scenarios, students engaged in a structured series of analytical and reflective enquiries. These encompassed identifying patient symptoms, formulating nursing diagnoses based on assessment, and conducting a mental status examination to evaluate the patient's condition. The mental status examination scrutinized objective (e.g., patient's general appearance and behavior) and subjective data (e.g., delusions, mood, and cognitive functioning). These discussions connected the observed clinical presentations and theoretical knowledge, fostering a comprehensive understanding of psychiatric assessments.

This study utilized two interactive pedagogical approaches: OSVE and role-play.

### Role-play sessions

Role-playing activities offered students practical experience cultivating vital competencies such as therapeutic communication, history-taking, and symptom management. The scenarios were designed to tackle clinical issues, including the management of hallucinations, delusions, suicidal thoughts, and aggressive behaviors. Each role-play session spanned 15 to 20 minutes, with students taking turns assuming the roles of nurses and simulated patients.

All scenarios were crafted following standardized guidelines and evaluated using rubrics developed and continuously improved by the course instructors. These rubrics assessed essential skills, including communication proficiency, critical thinking, and managing symptoms effectively. Instructors supervised the sessions to maintain consistency and to offer constructive feedback.

The diagnostic scenarios encompassed a range of psychiatric disorders at various stages, including acute symptoms, patients undergoing medication treatment, periods of remission, and emergency psychiatric situations. Furthermore, the scenarios distinguished between contexts involving hospitalized and non-hospitalized patients, providing comprehensive training applicable to various clinical environments.

Data were collected through in-depth semi-structured interviews using purposive sampling to recruit participants who experienced the phenomenon of interest. Individual interviews were conducted via Microsoft Teams using open-ended questions, each lasting 60 minutes. The interviews were conducted in Arabic, the participants' first language, to facilitate a clear explanation of their experiences. All interviews were audio recorded, and transcripts were transcribed verbatim after each interview. Transcripts were translated from Arabic to English, with a back translation conducted by both bilingual authors.

### Data analysis and management

The thematic analysis method was utilized within the constructionist paradigm [24]. In a constructionist perspective, meaning and experience are socially produced and reproduced rather than existing within individuals [25]. Therefore, data analysis followed the thematic analysis process outlined by Benner [21]:

**Step 1:** Researchers listened to each interview recording before transcribing and translating it. They then read the transcribed data multiple times to understand participants' experiences and documented their thoughts using reflective notes.
**Step 2**: Each researcher used the 'comments' function in Microsoft Word to generate initial codes, focusing on meaningful statements related to the research question.
**Step 3:** The coded data were reviewed and combined based on shared meanings to formulate themes.
**Step 4:** Researchers revised the themes and subthemes with the dataset.
**Step 5:** Both researchers organized the themes, defined them, and named them based on the participants' experiences.
**Step 6:** The final report was written based on the findings.

### Trustworthiness

To establish reliability, the study employed Lincoln & Guba's [26] four criteria: credibility, transferability, dependability, and confirmability. These criteria align with the sixth step of thematic analysis to enhance trustworthiness [27]. The research achieved credibility through prolonged engagement, detailed accounts of student experiences, collaborative analysis, peer review, and researcher reflexivity. Meticulous documentation of the research methodology and transparent data analysis procedures ensured the study's dependability. Transferability was demonstrated through verbatim interview transcripts, comprehensive descriptions of student experiences, collaborative analysis, peer consultation, reflexivity, and sampling techniques. Confirmability was maintained through a reflective diary documenting the researcher's ideas and responses.

The study's credibility was reinforced through prolonged engagement and detailed descriptions of student experiences. Thorough documentation of the research process and transparent data analysis methods contributed to the study's dependability. Verbatim interview quotes, detailed accounts of student experiences, and sampling approaches all enhanced the study's transferability. Maintaining a reflective journal documenting the researcher's thoughts and reactions ensured confirmability.

## RESULTS

### Demographic data

Table 1 shows that 20 nursing students were interviewed for this study. The participants' ages ranged from 21 to 26 years. They were single and represented various academic levels, including the 11^th^ level, 12^th^ level, and internships. All participants were from Jeddah and reported having a positive experience with the psychiatric clinical course. The research outcomes indicated that OSVE and role-play effectively developed psychiatric nursing competencies among students. For eight sessions, participants engaged in scenarios that replicated various psychiatric disorders, such as schizophrenia, bipolar disorder, and personality disorders. These scenarios depicted patients at different stages of their conditions, including acute episodes, those receiving medication, and individuals in remission. Additionally, cases involving emergency psychiatry and non-hospitalized patients were incorporated to create a realistic and varied training context.

**Table 1:** Participants' distribution according to their demographic background (*n* = 20)

Participants	Age	Marital status	Academic Level	Residence area	Enjoy studying psychiatric clinical course?
P1	22	Single	11	Jeddah	Yes
P2	23	Single	11	Jeddah	Yes
P3	23	Single	intern	Jeddah	Yes
P4	24	Single	intern	Jeddah	Yes
P5	24	Single	intern	Jeddah	Yes
P6	24	Single	intern	Jeddah	Yes
P7	26	Single	intern	Jeddah	Yes
P8	26	Single	9	Jeddah	Yes
P9	23	Single	12	Jeddah	Yes
P10	22	Single	12	Jeddah	Yes
P11	21	Single	12	Jeddah	Yes
P12	21	Single	12	Jeddah	Yes
P13	23	Single	9	Jeddah	Yes
P14	24	Single	9	Jeddah	Yes
P15	24	Single	9	Jeddah	Yes
P16	26	Single	12	Jeddah	Yes
P17	26	Single	11	Jeddah	Yes
P18	21	Single	9	Jeddah	Yes
P19	23	Single	12	Jeddah	Yes
P20	23	Single	12	Jeddah	Yes

After the OSVE sessions, students participated in instructor-led discussions, facilitating deeper analysis of symptoms, nursing diagnoses, and comprehensive mental status evaluations. The role-play activities further allowed students to practice and enhance their therapeutic communication and symptom management skills.

Instructor supervision was pivotal in maintaining the standardization of all sessions, ensuring alignment with the study's goals. Implementing structured rubrics offered a dependable framework for evaluating student performance and competencies.

### Themes

The findings of this study unveiled five overarching themes with corresponding subthemes that capture the essence of student experiences with simulation-based learning in psychiatric nursing (Table 2).

**Table 2:** Themes and subthemes from student experiences of osve and role-play in psychiatric nursing education

Themes	Sub-themes
1. Learning experience and professional development	
2. Learning challenges	1- Challenges in video simulations 2- Challenges in role play
3. Advantages of videos and role play simulations	1- Fostering empathy and enhancing communication. 2- Build confidence in a safe environment. 3- Readiness for clinical practice
4. Differentiating between video and role play simulation	1- OSCE/ OSVE: structured learning 2- Role plays simulations: active learning
5. Integration of OSVE and role play simulation	

#### Theme 1: learning experience and professional development

This theme highlights the profound impact of the psychiatric course on students, enhancing their knowledge and fostering a deeper enthusiasm for psychiatric nursing by interacting with patients and understanding mental illnesses. A participant stated, "*Engaging with patients and gaining a deeper understanding of mental illness were rewarding aspects of the course, leaving a lasting impression on my nursing education journey*" (P2).

A progression from initial unfamiliarity to a more profound understanding and acknowledgment of mental health care characterizes the transformation in education encountered by students studying psychiatric nursing.

One student articulated, "*Before this course, I had little understanding of psychiatric conditions. Now, I have a deeper appreciation and respect for what patients go through*" (P5).

Students shared a notable change in their attitudes towards mental health and patients with mental illness, transitioning from fear and misunderstanding to empathy and respect.

A participant reflected, "*I was afraid at first, but as I interacted with patients and learned about their experiences, my outlook completely shifted*" (P10).

All respondents conveyed that their participation in psychiatric clinical rotations delivered a distinctive and innovative educational opportunity unlike any other nursing course.

A participant disclosed, "*Addressing the psychological dimension was a fresh subject separate from the emphasis on physical ailments in other modules*" (P1).

Most respondents noted that engaging in simulated patient role-playing was a novel encounter for them, as one student remarked, "*Utilizing role plays for the first time proved to be advantageous in gaining insights and readiness for patient interaction*" (P7).

Furthermore, exposure to psychiatric nursing also inspired new career aspirations and fostered positive attitudes toward mental healthcare.

A participant said, “*The course has deepened my interest in psychiatric nursing, and the impact we can have in psychiatric settings has led me to contemplate a career in this field*” (P1).

Moreover, participants reported that psychiatric clinical rotation fosters the development of critical thinking and problem-solving skills for nursing practice.

A participant said, "*I learned to think critically about patient care and solve issues, which will be used across nursing specialties*" (P7). Similarly, another participant added, “*The course underscored the importance of treating each patient as a unique individual with specific needs. I remember assisting a patient who was hesitant to share his experiences, which taught me the importance of building trust and providing compassionate care*" (P6).

#### Theme 2: learning challenges

The theme addresses students' challenges, particularly with language in video simulations and role-plays. Participants highlighted various aspects of these barriers and emphasized how they affect their learning journey.

##### Sub-theme 1: challenges in video simulations

Students recognized challenges like initial apprehension and uncertainty when interacting with psychiatric patients in video simulations. Many students expressed difficulties with language, especially when the videos featured English content. This involved struggles with understanding accents, poor audio quality, and a lack of Arabic resources. Participants explained that the complexity of the language used in video simulations was cited as a major issue, leading to a lack of comprehension and impeding their ability to grasp and retain information because English is not their primary language.

One participant reported, “*The language barrier, given that English is not my first language, made it hard to comprehend certain words or phrases*” (P1).

The rapid content presentation made it difficult for participants to absorb information from video simulations.

One participant stated, “*The competencies need to be administered more through increasing the video numbers and increasing the time for the OSVE or repeating the video to catch the information*” (P4).

Some participants noted that, despite language not being a barrier, the need for multiple viewings of videos is important to comprehend the complexity of the video content. Another student reported, “*The language was not a barrier; however, repeating the video multiple times proves more beneficial*” (P6).

##### Sub-theme 2: challenges in role play

All participants encountered challenges in the role-play exercises, such as uncertainties about role-plays and the necessity for increased practice. Despite these challenges, participants found role-playing to be more beneficial. Most participants reported that accurately depicting psychiatric scenarios was emotionally challenging, while others found it easier to detach themselves emotionally.

One participant stated, “*Imitating patient hallucinations was challenging*” (P9).

Many students initially expressed hesitance to engage in role-plays due to their lack of familiarity with acting and limited experience.

One participant articulated, "*I was initially hesitant to initiate the role-play because of a lack of knowledge on how to commence it; however, over time, I acquired the skills to perform*" (P7).

These challenges also encompassed complexities in replicating specific psychiatric conditions and the importance of thorough preparation.

Another participant highlighted, "*Replicating patient hallucinations proved to be a more challenging task, yet it enhanced my comprehension of the patient's circumstances. It serves as a beneficial tool for educational purposes, as active engagement fosters learning*" (P9).

#### Theme 3: advantages of videos and role-play simulations

This theme highlights the advantages of using videos and role-play simulations in psychiatric nursing education. Participants emphasized that these educational tools significantly improved their understanding, competence, and confidence in dealing with mental health conditions.

##### Sub-theme 1: fostering empathy and enhancing communication

All participants noted that these simulations helped cultivate empathy by allowing them to see things from the patient's perspective.

A student shared, "*A specific moment emphasized the importance of effective communication, empathy, and cultural competence in psychiatric nursing practice. I learned the crucial role of communication in patient care from a therapeutic communication video simulation*." (P8). Participants mentioned that role-play enhanced empathy by immersing them in the roles of patients and healthcare providers, improving their communication skills, patient interaction, and symptom management.

A participant mentioned, "*I learned to be present with the patient rather than bombard them with questions*" (P12).

Most participants found that consistent communication strategies learned and applied in practice improved patient interactions.

A student recounted a participant's experience, "*I had a patient with schizophrenia who was confused, and our communication with him was calming. The video simulation was helpful, and I watched YouTube videos to learn. When I encountered a similar situation in the hospital, I applied what I had learned*." (P3).

##### Sub-theme 2: building confidence in a safe environment

All participants reported that simulations boosted their confidence and helped them develop a stronger professional identity as nurses.

A participant mentioned, "*The course gave me the confidence to handle challenging situations and feel more secure in my role as a nurse*" (P8).

Many participants initially felt nervous during role-plays but gained confidence as they engaged with patients and deepened their understanding of mental illness.

A participant shared, "*Spending time with patients became interesting. They were cooperative, helpful, and kind*."

Most participants found that simulation-based learning provided realistic exposure to various psychiatric conditions, enhancing their confidence and competence in patient care.

A student stated, "*Role play helped me improve my skills and build trust in interacting with patients*." (P9).

Many participants mentioned that practicing in a controlled environment through simulations helped them gain confidence in handling complex clinical situations and interacting with patients.

"*I was hesitant to start the role play initially, but I learned to engage more naturally over time*." (P10).

Participants also highlighted that role-play simulations offer a safe space to practice with peers, refining their abilities without fear of mistakes.

A participant mentioned, "*It is a safe environment to practice before working with actual patients*." (P3). Another participant stated, "*Role plays prepared me to communicate effectively and put patients at ease*" (P7).

##### Sub-theme 3: readiness for clinical practice

All participants found that video and role-play simulations bridged theoretical knowledge with practical scenarios, preparing them for real-life clinical situations.

A participant mentioned, "*The OSVE assessments provide a structured platform to showcase my competencies in various clinical scenarios, helping me apply theoretical knowledge to practical settings*." Another participant stated, "*Role plays equipped me for interacting with patients in clinical settings*." (P12).

Participants emphasized that role-play sessions with simulated patients simulate real-life psychiatric encounters effectively.

A student shared, "*Video simulations have been a valuable educational tool, equipping me with essential skills to manage scenarios like interacting with patients experiencing hallucinations in clinical practice*." (P1). Another participant added, "*Role plays simulated real clinical scenarios, familiarizing me with patient interactions*."

Feedback from instructors and peers was highlighted as beneficial in improving communication skills and therapeutic approaches.

A participant reflected, "*Instructors' advice to review and analyze scenarios thoroughly has been particularly helpful in understanding patient interactions and nursing interventions*."

#### Theme 4: differentiating between video and role-play simulation

The distinction between OSCEs, OSVEs, and role-play in psychiatric nursing education reveals critical insights across several sub-themes.

##### Sub-theme 1: OSCE/OSVE: structured learning

OSCEs enhanced students' clinical insights while promoting equity and accurate assessment. In the OSVE group, participants emphasized the structured nature of their learning experiences.

One participant mentioned, “*The use of videos helps us vividly imagine what it might be like for a patient to undergo such an episode. This approach enhances our understanding of hallucinations and informs our approach to interacting with patients who experience them*.” (P4).

Furthermore, they reported that OSVE provides a standardized environment to observe a comprehensive view of patients’ symptoms and behaviors. Participants illustrated the opportunity to analyze videos, apply accurate nursing interventions, and identify diagnoses.

A participant shared, "*I could observe various psychiatric presentations in a controlled environment. My observational skills, understanding, and identifying the different symptoms were sharpened by OSVE*" (P7).

All participants underscored the importance of OSCEs in fostering their development of competencies for effective patient care, such as taking history, clinical examination, and ethical practice.

One participant articulated this developmental process, stating, “*In the first week, starting with history-taking competence role-play was difficult, and in the end, it became easier and more familiar, making me more comfortable interacting with patients*." (P6)

All participants in the OSCE group consistently emphasized the value of a structured and standardized evaluation of OSCEs in practicing and validating critical skills.

“*The OSCE of ethical consideration and the correct way to treat patients helped me understand how to treat and be involved with them*.”

##### Sub-theme 2: role-play simulations: active learning

The participants in the role-play groups reported that role-play offers a more flexible and dynamic approach to learning and explores various aspects of patient care in a less formal setting.

Participants reported, “*The role-play helped me be involved with the patient... acknowledge that the symptoms sometimes make it difficult to be concerned*” (P7).

Furthermore, participants explained that the flexible role-play environment enhances their learning experience.

A student stated, “*I had to read and know how to act for the role-play; it was more helpful*” (P9).

Participants emphasized that role-play provided a hands-on approach to understanding and managing symptoms, such as hallucinations and delusions.

A participant illustrated, "*By assuming both caregiver and patient roles, participants reported gaining insights into these symptoms' emotional and psychological impact, as the participant reported, 'being with patient experiences in role-play makes me more involved within patient worlds*.” (P6).

#### Theme 5: integration of OSVE and role-play simulation

All participants recommended a comprehensive approach by integrating OSVEs and role-play simulations, which can enhance their readiness for clinical practice. This integration equips them with the knowledge, skills, and confidence necessary for psychiatric nursing.

One participant explained, “*The integration of simulation methods has significantly enhanced my preparedness to handle real-world situations in psychiatric nursing. These experiences have not only provided me with practical skills and knowledge but have also instilled confidence in my ability to deliver high-quality care to patients with mental health concerns*.”

Participants reported the benefit of utilizing both simulations to understand the illness’s characteristics.

A student explained, “*The first role-play was challenging because this was the first time I learned about the patient. The video helped me understand the symptoms and how to manage them, and after that, I can live through these experiences to do the role-play*.”

Others emphasized the value of visual learning tools, such as video simulations, in reinforcing their learning experience.

One participant noted, “*By combining these methods, I can benefit from the visual learning experience provided by video simulation while actively engaging in practical scenarios through role-play with simulated patients. This dual approach would allow me to apply theoretical knowledge in realistic situations, enhancing my understanding and retention of psychiatric nursing concepts*.” (P1).

## DISCUSSION OF THEMES

The first theme of *learning experience and professional development* encompasses the significant impact of simulation-based learning on psychiatric nursing students. This educational approach enhances knowledge, fosters a more profound understanding, and promotes respect for mental healthcare.Krumwiede [28] highlighted that simulation in nursing education improves students' clinical skills and knowledge retention, leading to increased confidence and enthusiasm in their abilities. McCaughey & Traynor [29] emphasized that simulation-based learning in psychiatric nursing enhances students' understanding of mental health conditions and therapeutic communication skills. Ferr *et al*. [30] also observed a positive shift in nursing students' attitudes towards mental health, moving from fear and stigma to empathy and admiration. Hands-on experiences with patients during simulations, as noted by Alrashidi *et al*. [31], help students develop a more empathetic approach to care. Akbari *et al*. [5] reported that simulation experiences in psychiatric settings inspire students to pursue careers in mental health fields, recognizing the impact they can have. Khalil, Hantira, and Alnajjar [32] found positive simulation experiences could increase undergraduate students' interest in psychiatric nursing careers. However, Bø *et al*. [33] raised concerns about the cost-effectiveness of simulation-based education and questioned whether the benefits justify the expenses. Kim *et al*. [34] found that while simulation is beneficial, its long-term impact on skill and knowledge retention might not be significantly higher than traditional teaching methods. Some students, as highlighted by Abdalrahim [35], may not respond well to simulation-based learning and may experience increased anxiety and stress. Similarly, Jallad [36] noted that the effectiveness of a simulation could vary based on the quality of scenarios and facilitation provided.

The second theme, *learning challenges in video simulations and role-play*, delves into the challenges students face, particularly with the language used in video simulations and role-plays. It underscores the significant impact of these barriers on their learning journey and emphasizes the need for adaptations to improve learning outcomes. Students often encounter apprehension and uncertainty when interacting with psychiatric patients, compounded by language barriers in video simulations. Many participants struggled with understanding accents, had poor audio quality, and lacked resources in their native Arabic language. This language complexity impeded their comprehension and retention of information, especially since English was not their first language.

One participant mentioned, “*The language barrier, as English is not my first language, made it difficult to understand certain words or phrases*” (P1).

The rapid delivery of content in video simulations posed another significant challenge. Participants expressed the need for more time and repetition to grasp the material.

One participant suggested, “*The competencies should be delivered more through increasing the number of videos and extending the time for OSVE or replaying the video to capture the information*” (P4).

Even when language was not a barrier, students found multiple viewings beneficial in understanding the complexity of the content.

One student stated, “*Language was not a barrier; however, watching the video multiple times proved more beneficial*” (P6).

Recognizing the challenges students face in video simulations is essential for optimizing teaching methods in psychiatric nursing. Language barriers are prevalent, highlighting the need for inclusivity in educational resources. Bandura’s Social Learning Theory [37] suggests that learning occurs through observation, imitation, and modeling, emphasizing the importance of clear language and simulation content for students' learning and performance in real-life scenarios.

The third theme, *advantages of videos and role-play simulations*, highlighted the significant benefits of using videos and role-play simulations in psychiatric nursing education. Participants emphasized that these tools greatly improved their understanding, competence, and confidence in dealing with mental health conditions.

### Fostering empathy and enhancing communication

The role of empathy in psychiatric nursing is crucial, as noted by the participants. Simulations were seen as instrumental in fostering empathy by allowing students to view scenarios from the patient's perspective.

One student shared a poignant experience: "*A specific moment highlighted the importance of effective communication, empathy, and cultural competence in psychiatric nursing practice. I learned about the pivotal role of communication in patient care through a therapeutic communication video simulation*." This underscores the idea that empathy can be developed through experiential learning and reflection [38].

### Enhancing communication skills

Simulated patient roles provided a valuable platform for practicing and refining communication strategies. After applying these strategies in practice, participants reported significant improvements in their patient interactions. For example, one student mentioned how a video simulation helped them learn from YouTube and apply those skills effectively in a real hospital setting [39].

The advantages of video and role-play simulations in psychiatric nursing education are diverse and contribute to the holistic development of nursing students. These tools align with experiential learning theories, emphasizing the importance of concrete experiences and reflective observation in learning [40]. Students undergo a transformative learning journey by engaging in simulations, developing empathy, and refining their communication skills essential in psychiatric nursing.

Simulations offer a safe environment for students to practice and learn from mistakes without real-world consequences, enhancing their professional identity and readiness for clinical practice. This aligns with Bandura's Social Learning Theory [41], which suggests that learning occurs through observation, imitation, and modeling. Students can build the skills and confidence to handle clinical situations effectively through simulated scenarios.

Consistent feedback from instructors and peers supports the development of communication and therapeutic skills, fostering continuous improvement and competence in psychiatric nursing. This iterative process of practice and feedback is crucial for building the confidence and skills necessary to deliver high-quality patient care.

Contrasting structured and active learning approaches, the distinction between OSCE/OSVE and role-play simulations in psychiatric nursing education highlights the unique advantages of both approaches. OSCEs and OSVEs provide a structured framework that ensures standardized assessment and development of critical clinical competencies. This approach is particularly beneficial for enhancing observational skills, applying theoretical knowledge in a controlled environment, and fostering a comprehensive understanding of patient care practices. In contrast, role-play simulations offer a more flexible and dynamic learning environment that emphasizes active engagement and hands-on experiences. This approach allows students to immerse themselves in the patient's world, fostering empathy and a deeper understanding of psychiatric symptoms' emotional and psychological impacts. The active preparation required for role-play simulations ensures that students fully engage in learning, leading to a personalized and impactful educational experience. Both approaches are complementary and provide a well-rounded educational experience for nursing students. While OSCEs and OSVEs offer the necessary structure and standardization for developing essential clinical skills, role-play simulations provide the flexibility and immersion required to foster empathy and active learning. These methods equip nursing students with the comprehensive skills and insights required for effective psychiatric care. Integrating OSVEs and role-play simulations in psychiatric nursing education offers a multifaceted approach that significantly enhances students' readiness for clinical practice. Participants emphasized that combining these simulation methods equips them with the practical skills, knowledge, and confidence necessary to provide high-quality care to patients with mental health concerns.

One participant highlighted, “*The integration of simulation methods has significantly enhanced my preparedness to handle real-world situations in psychiatric nursing. These experiences have not only provided me with practical skills and knowledge but have also instilled confidence in my ability to deliver high-quality care to patients with mental health concerns*."

This finding is consistent with Cortés-Rodríguez *et al*. [41], who suggested that combining different simulation methods can bridge the gap between theoretical knowledge and practical applications, enhancing clinical competence and confidence. Participants noted that using both OSVEs and role-play simulations helped them understand psychiatric illnesses better.

One student shared, "*The first role-play was challenging because it was my first encounter with the patient. The video helped me grasp the symptoms and management strategies, making the role-play more meaningful*."

Mitra and Fluyau [42] support this idea, stating that integrating visual learning tools with interactive simulations deepens students' understanding of psychiatric symptoms and response strategies. The combination of video simulations and role-play was found to reinforce learning experiences.

A participant mentioned, "*By combining these methods, I can benefit from visual learning through video simulations and engage in practical scenarios through role-play with simulated patients. This dual approach allows me to apply theoretical knowledge in realistic situations, enhancing my understanding of psychiatric nursing concepts*."

Qu *et al*. [43] also agree, stating that a dual-method approach caters to different learning styles, improving cognitive retention and application of knowledge in clinical settings. However, Gkintoni *et al*. [44] caution that combining different simulation methods may overwhelm some students, suggesting a phased integration approach to ensure thorough understanding before incorporating multiple methods.

Although the research was conducted in Saudi Arabia and the population's mother tongue is Arabic, the fundamental skills addressed, including therapeutic communication and empathy, have universal significance. The scenarios can be adjusted to incorporate local cultural subtleties and patient characteristics to implement this approach in different environments. The global applicability of this technique can be further improved by rendering OSVE materials in local languages and ensuring that they align with regional linguistic and societal standards [45].

Using OSVE and role-play as pedagogical tools provides a robust framework for bridging theory and practice in psychiatric nursing education. OSVE, with its pre-recorded or instructor-created video scenarios, allowed students to explore diagnostic features of psychiatric disorders in a structured and reflective manner. The guided questions following each scenario—focused on symptoms, nursing diagnoses, and mental status examinations—ensured that students developed critical assessment skills while connecting theoretical concepts with clinical realities. The introduction of a variety of diagnostic scenarios, encompassing different stages of psychiatric disorders, significantly deepened students' comprehension of symptomatology and nursing interventions.

Furthermore, the role-play sessions complemented the observational learning in OSVE by providing an interactive environment for students to practice and refine essential skills such as therapeutic communication and crisis management. Role-play activities enhanced the OSVE experience by allowing students to actively practice therapeutic communication and navigate intricate psychiatric symptoms. Using structured rubrics guarantees that evaluations are objective and consistent with course competencies. Instructors are crucial in supervising OSVE and role-play sessions, ensuring scenario standardization and offering constructive feedback to enhance skills.

These pedagogical approaches equip students to manage complex psychiatric cases and consider cultural and clinical diversity, rendering the outcomes applicable across various educational and healthcare environments. Future investigations could focus on integrating personality assessments of participants to mitigate subjectivity in response evaluations and yield more profound insights into the educational experience.

In conclusion, integrating OSVEs and role-play simulations offers a robust framework for psychiatric nursing education, providing students with a deeper understanding and practical skills for clinical practice. Careful implementation and phased integration can prevent cognitive overload, allowing students to benefit fully from the combined approach. These insights emphasize the essential role of simulations in preparing nursing students for psychiatric care challenges and nurturing empathetic, confident, and effective healthcare providers. They also suggest a pathway for developing educational tools that cater to students' diverse linguistic backgrounds, enhancing their learning experiences and competencies in psychiatric nursing.

## CONCLUSION

Integrating role-play and OSVEs in psychiatric nursing education offers a comprehensive and multifaceted approach to learning. These methods help students bridge theoretical knowledge with practical application, preparing them for clinical practice. Visual and interactive elements enhance students' understanding of psychiatric conditions, foster empathy, and improve communication skills. While each approach has advantages and limitations, their combination provides a well-rounded learning experience, equipping students with the confidence and competence to handle real-world clinical situations effectively.

### Recommendations for effective psychiatric nursing education

Based on student feedback, educational institutions can enhance the benefits of incorporating role-plays and OSVEs by following these recommendations:

- Introduce OSVEs to establish a structured understanding of psychiatric conditions, followed by role-play to reinforce knowledge dynamically.
- Ensure video simulations are accessible in multiple languages with clear audio and subtitles to accommodate diverse linguistic backgrounds.
- Provide regular feedback sessions from instructors and peers to help students refine their skills and therapeutic approaches.
- Start with simpler role-play scenarios and gradually increase complexity to build students' confidence.
- Allocate resources for high-quality video production and trained facilitators to support effective simulation implementation.
- Aim for high fidelity in role-play scenarios by involving standardized patients or actors to enhance realism.
- Encourage peer collaboration in role-plays to improve learning outcomes.
- Evaluate the effectiveness of simulation methods through student feedback and performance assessments to adapt the curriculum accordingly.

### Nursing implications

- Educators should design educational simulations that accommodate varying linguistic proficiency levels and processing speeds to optimize learning experiences for all students.
- To address linguistic diversity, provide multilingual resources such as captioning, translations, and recordings in different languages.
- Ensure audio quality is clear and understandable to assist students with different accents in auditory learning.
- Allow flexible viewing options for students to revisit lessons at their own pace.
- Provide supplementary materials in students' native languages to support comprehension and retention of information.

By incorporating these considerations and adapted methodologies, higher education institutions can cultivate psychiatric nursing proficiency among diverse learners, preparing graduates to deliver compassionate and evidence-based care to individuals with mental health issues. Continuous evaluation through performance indicators and feedback allows for ongoing improvement of integrated simulation practices.

## Personal thanks

The authors express their gratitude to all students for their active participation in the study and attendance at the interviewing sessions, as their contributions were essential to completing this research.
